# Evaluating leflunomide and methotrexate combination vs. monotherapy in rheumatoid and psoriatic arthritis

**DOI:** 10.55730/1300-0144.6010

**Published:** 2025-03-24

**Authors:** Mete KARA, Gülay ALP, Mete PEKDİKER, Sertaç KETENCİ, Annamaria PORRECA, Haluk CİNAKLI

**Affiliations:** 1 Department of Rheumatology, İzmir City Hospital, İzmir, Turkiye; 2 Department of Rheumatology, Faculty of Medicine, Uşak University, Uşak, Turkiye; 3 Department of Rheumatology, Faculty of Medicine, Mustafa Kemal University, Hatay, Turkiye; 4 Department of Rheumatology, Faculty of Medicine, Ondokuz Mayıs University, Samsun, Turkiye; 5 Department of Human Sciences and Promotion of the Quality of Life, San Raffaele, Rome, Italy; 6 Unit of Clinical and Molecular Epidemiology IRCCS San Raffaele Roma, Rome, Italy; 7 Department of Rheumatology, Kırklareli Educational and Research Hospital, Kırklareli, Turkiye

**Keywords:** Combination therapy, leflunomide, methotrexate, monotherapy, psoriatic arthritis, rheumatoid arthritis

## Abstract

**Background/aim:**

Methotrexate (MTX) and leflunomide (LEF) are conventional synthetic disease-modifying antirheumatic drugs (DMARDs) commonly used for treating rheumatoid arthritis (RA) and psoriatic arthritis (PsA), either as monotherapies or in combination. This study aimed to compare the adverse effects (AEs) and efficacy of combined use of MTX plus LEF with monotherapy in RA and PsA patients.

**Materials and methods:**

This study included 528 patients (385 RA and 143 PsA) with at least 6 months of follow-up. Disease activity was assessed using DAS-28 CRP and DAPSA, and treatment-related AEs were classified based on specific MedDRA categories.

**Results:**

The cumulative incidence of AEs in patients with RA treated with MTX, LEF, and MTX plus LEF was 23.8%, 28.2%, and 19.2%, respectively; in PsA patients, 18.1%, 30%, and 23.3%, respectively. None of the groups were superior to each other in terms of general AEs between monotherapy and in combination. LEF monotherapy in RA was associated with more neurological AEs and hypertension. Compared to monotherapy, MTX plus LEF demonstrated greater reductions in disease activity, a more substantial decrease in glucocorticoid doses, and lower utilization of biological/targeted DMARDs (b/tsDMARDs) in both RA and PsA. In univariate analysis, MTX dosage, initial DAS28 CRP, and b/tsDMARD initiation were predictors of low disease activity (LDA) in RA. In multivariate analysis, MTX dosage (95% CI 1.02–1.59, OR = 1.27, p = 0.031) and initial DAS28-CRP (95% CI 2.14–10.90, OR = 4.38, p < 0.001) were found to be independently associated with LDA in RA. In PsA, the factors associated with LDA were initial DAPSA and disease duration in univariate analysis. Only disease duration was found to be an independent predictor in multivariate analysis (95% CI, 1.02–1.38, OR = 1.17 p = 0.039).

**Conclusion:**

Adding LEF to MTX or vice versa may serve as a valuable, safe, and effective alternative in situations where b/tsDMARD therapy is challenging.

## 1. Introduction

Rheumatoid arthritis (RA) is a chronic autoimmune and multisystemic disease primarily affecting synovial joints and extraarticular structures in a significant portion of patients, increasing mortality and morbidity. The prevalence of RA was estimated to be approximately 0.24% in 2010 [1] and considering the chronic course of the disease, a significant disease burden for the patient and society [2,3]. RA is a highly progressive disease and can rapidly lead to permanent disability within two to three years of diagnosis without treatment [4–6]. It is crucial to initiate treatment with disease-modifying antirheumatic drugs (DMARDs) as soon as RA is diagnosed, as delay results in significantly greater radiographic damage within 5 years. The European Alliance of Associations for Rheumatology (EULAR) recommendation suggests no superiority for any combination of conventional synthetic (cs) DMARD over monotherapy [5]. However, there is still no consensus on the most effective combination of csDMARDs for RA management [3].

Similar to RA, psoriatic arthritis (PsA) is a form of peripheric arthritis affecting the joints, entheseal regions, spine, skin, and nails [7]. Even a 6-month delay in diagnosis and initiation of antirheumatic treatment can cause long-term damage and disability in PsA [8]. Growing evidence of the pathogenesis of these diseases has led to significant improvements in therapeutic options, especially in the biological agent area, within the last decade. However, for the biologic or target synthetic (b/ts) DMARDs, there are few studies showing their efficacy over methotrexate (MTX) monotherapy with temporal glucocorticoids (GCs) or csDMARDs. Still, b/ts DMARDs have more adverse effects (AEs), such as infectious events or malignancies. Therefore, the effective and safe use of csDMARDs is crucial [9].

MTX and leflunomide (LEF) are the most commonly used csDMARDs and have different mechanisms of action. They can be used alone or in combination with both csDMARD and b/tsDMARD at any stage of RA management [10].

AEs are among the biggest concerns in the combined use of MTX and LEF. Even when these drugs are used alone, significant AEs are reported. For example, 60% of patients with RA using MTX experience at least one AE (i.e. nausea, vomiting, elevated liver enzymes, alopecia, headaches, etc.), and complaints may persist in continuation [11]. Similar AEs are also reported for LEF [12]. Although there is positive data regarding the use of MTX and LEF alone, different results are observed in terms of the efficacy and AEs of these drugs when used in combination. MTX plus LEF combination therapy has the same effectiveness in treatment-naive patients, and the patients’ adherence to the drug is similar despite different AEs and tolerances [11]. Although studies show that combination therapy is comparable in terms of AEs in RA [13], there is limited data for PsA [7].

Therefore, the primary objective of this study was to compare the cumulative incidence of AEs amongst LEF alone, MTX alone, and the combination of MTX plus LEF (combination therapy) regimens in RA and PsA patients. The secondary objectives were to investigate the effects of these different regimes on the initiation of steroid tapers, b/tsDMARDs, and disease activity.

## 2. Materials and methods

### 2.1. Study design

This retrospective observational study was conducted between January 2020 and June 2022 at the Department of Rheumatology at the University of Health Sciences İzmir Bozyaka Education and Research Hospital. It included patients with more than 6 months of follow-up who were diagnosed with RA according to the 2010 Rheumatoid Arthritis Classification Criteria by the American College of Rheumatology/EULAR (ACR/EULAR) [14] and those who were accepted as PsA according to the Classification of Psoriatic Arthritis Criteria [15].

Patients were excluded if they a) were not receiving treatment due to difficulty in prescription (i.e. challenges in accessing a rheumatologist), b) did not have regular follow-ups, c) stopped drug treatment, d) used other drugs than MTX, LEF, or MTX plus LEF such as hydroxychloroquine, sulfasalazine, and steroids, e) had malignancies, f) had increased liver enzymes, g) had renal dysfunction h) were PsA patients with only axial involvement, and i) were pregnant and lactating. The 2019 EULAR RA and PsA treatment recommendations were followed for the treatment of patients diagnosed with RA and PsA [16,17]. We aimed to achieve low disease activity (LDA) using the Disease Activity Score C-Reactive Protein (DAS28-CRP) and Disease Activity in Psoriatic Arthritis (DAPSA) for RA and PsA, respectively. The treatment of the patients was decided according to the follow-up physician’s evaluation, considering their age, comorbidity, kidney failure, medication adherence, initial disease activity, and risk factors. MTX plus LEF combination therapy or b/tsDMARD combination were started in all patients in case of inadequate monotherapy; MTX and LEF were not started simultaneously in any patient.

Patients were grouped retrospectively according to the treatments they received, divided into MTX monotherapy, LEF monotherapy, and MTX plus LEF combination therapy, and efficacy and AEs were compared between groups. The reporting of this study conforms to the strengthening the reporting of observational studies in epidemiology (STROBE) statement [18].

### 2.2. Demographic, clinical variables, and adverse effects

The demographic characteristics of the patients, their usage of medication, smoking status, and comorbidity data were noted from the electronic patient registration system. All comorbidity data have been processed in accordance with the classification of diseases described by Charlson [19]. The DAS28-CRP [20] was used to evaluate disease activity for RA and DAPSA [21] for PsA. RA patients were grouped as having LDA if their DAS28-CRP was ≤3.2, and PsA patients were grouped as having LDA if their DAPSA was ≤14.

AEs were registered at each study visit by the physician and coded according to the Medical Dictionary for Regulatory Activities (MedDRA) coding system [22]. Routine toxicity screening was performed at baseline and follow-up visits. Safety features were recorded at regular, frequent intervals (initially monthly, then every 3 months). At each patient visit, a complete blood count, biochemical profile, urinalysis, and detailed anamnesis were recorded, and these records were reviewed retrospectively. In case of AEs or suspected intolerance, extra laboratory tests were performed on each patient by the same physician, or the dose of the corresponding drug was reduced or discontinued, or both. Serious AEs are defined as needing hospitalization, serious infectious events (such as hepatitis B reactivation, tuberculosis, disseminated varicella-zoster, fungal infections, or requiring intravenous antibiotics), permanent organ damage, or death.

### 2.3. Statistical analysis

Both visual (histograms and probability plots) and analytical methods (Kolmogorov-Smirnov test) were used to check whether the variables had a normal distribution. The categorical variables were represented as percentages and frequencies, and the continuous variables were represented as mean ± standard deviation (SD) or median interquartile range (IQR) values. Categorical variables were assessed by chi-squared test if n ≥ 5 and Fisher’s exact test otherwise. One-way analysis of variance (ANOVA) with the Bonferroni post hoc test were used to compare three independent groups with normal distribution, and the Kruskal–Wallis test was used to compare three groups of nonnormally distributed data. Univariate and multivariate logistic regression models were used to evaluate the factors associated with LDA for RA and PsA. We used a stepwise regression approach based on the Akaike information criterion (AIC) to select the most relevant confounding variables. This method iteratively added or removed variables to minimize the AIC to balance model accuracy and complexity. The collected data were analyzed using the R environment version 4.2 and the IBM SPSS version 25.0 (IBM Corp., Armonk, NY, USA).

## 3. Results

### 3.1. Baseline demographic and clinical characteristics

A total of 528 (385 RA and 143 PsA) patients (in RA: MTX, n = 248; LEF, n = 85; MTX plus LEF, n = 52; in PsA: MTX, n = 83; LEF, n = 30; MTX plus LEF, n = 30) were included. Of all the patients, 77.5% were female, the mean age was 53.8 ± 13.9 years, the age at diagnosis was 48.6 ± 14.1, the median disease duration was 3 years (IQR = 5), and the mean follow-up period was 30 ± 18 months. The mean follow-up period was 29.4 months for RA patients and 27.3 months for PsA patients, with no statistically significant difference between them (p = 0.752). When RA and PsA were compared, PsA patients had a lower age at diagnosis and a higher rate of MTX plus LEF combination therapy. The smoking status in the combination therapy group was higher in both RA and PsA patients. The number of rheumatoid factor and/or anticitrullinated protein antibody-positive patients in RA was 249 (64.7%) and 5 (3.5%) in PsA patients. All patients were given folic acid to minimize MTX AEs and received a mean of 10 ± 2.2 mg of folic acid supplements weekly. RA patients had more comorbidities, and other disease clinical and activity characteristics were similar between groups (Table 1).

### 3.2. Treatments

In combination therapy, the mean MTX dose was 14.6 ± 2.9 mg/week, and LEF dose was 114.8 ± 32.3 mg/week in RA, while in PsA, the mean MTX dose was 14.1 ± 3.2 mg/week, and LEF dose was 116.6 ± 33.5 mg/week. Neither group was superior to the other (Table 2 and 3). In both RA and PsA patients, the initial steroid dose was higher in the combination group. The difference between the initial steroid dose and the final steroid dose (Δ steroid) was found to be significantly higher in the MTX plus LEF combination group than in the MTX and LEF monotherapy groups (all p-values < 0.001). In both RA and PsA, LEF monotherapy was significantly more preferred in patients with advanced age. Additionally, MTX monotherapy was more favored in women in the RA group, while LEF monotherapy was more preferred in patients with comorbidities in PsA. In the treatment groups, MTX and LEF dosages were significantly higher in the monotherapy group than in the combination groups. Drug doses in combination therapy were similar in RA and PsA patients.

### 3.3. Safety

The cumulative incidence of AEs in patients with RA using MTX, LEF, or MTX plus LEF was 23.8%, 28.2%, and 19.2%, respectively, but there was no statistically significant difference (p = 0.47) (Table 4). Serious AEs were observed in 5 patients (three in RA and two in PsA), including acute interstitial pneumonia in one patient receiving MTX therapy and hepatotoxicity in one patient receiving LEF treatment. In the group receiving MTX plus LEF combination therapy, cytomegalovirus pneumonia was observed in one patient, *Klebsiella pneumoniae* was observed in one patient, and pancytopenia was observed in one patient. For specific AEs, no significant differences were observed between treatment groups. In the RA group, patients using LEF monotherapy had more neurological AEs (p = 0.019) and newly developed hypertension (p = 0.014) (Table 4). The cumulative incidence of AEs in patients with PsA using MTX, LEF, or MTX plus LEF was 18.1%, 30%, and 23.3%, respectively, but there was no statistically significant difference between groups (p = 0.38). In the PsA group, there was no significant difference between specific AEs with respect to the treatments (Table 5).

### 3.4. Efficacy

In the combination group, the baseline mean ± SD DAS28-CRP was 5.15 ± 1.14, and the final DAS28-CRP was 1.78 ± 0.57 in RA patients (Table 2), while the baseline mean DAPSA was 29.76 ± 5.18, and the final DAPSA was 6.31 ± 2.68 in PsA patients (Table 3). The mean DAS28-CRP score in RA was significantly higher in the combination group at baseline. Active disease at baseline was similar in both RA (95.6%) and PsA (93.7%) patients. At the final visit, the prevalence of active disease was 4.2% for RA and 7.7% for PsA, with no significant difference observed between the groups. In both RA and PsA patients, there was a significant decrease in disease activity scores in the combination group compared to monotherapy (Figure). Due to inadequate response to csDMARD treatment in PsA and RA patients, the cumulative incidence of bDMARD and b/tsDMARD initiation was less in the MTX plus LEF combined group compared to the MTX and LEF monotherapy groups.

In univariate analysis, MTX dosage, initial DAS28 CRP, and b/tsDMARD initiation were predictors of LDA in RA. In multivariate analysis, MTX dosage (95 % CI 1.02–1.59, OR = 1.27, p = 0.031) and initial DAS28-CRP (95%CI 2.14–10.90, OR = 4.38, p < 0.001) were found to be independently associated with LDA (Table 6).

In PsA, the factors affecting LDA were initial DAPSA and disease duration in univariate analysis. Only disease duration was found to be an independent predictor in multivariate analysis (95% CI 1.02–1.38, OR = 1.17 p = 0.039) (Table 7).

## 4. Discussion

The combined use of MTX plus LEF showed a significant improvement in disease activity compared to either drug alone, although there was no difference in AEs when used at appropriate doses. Furthermore, the addition of LEF to MTX or vice versa had a steroid-tapering effect and significantly reduced b/tsDMARD initiation in both RA and PsA patients.

In our study, while the MTX plus LEF group was more effective than MTX and LEF monotherapy in RA patients, there was no significant difference regarding AEs. The effectiveness of MTX plus LEF combination was higher compared to MTX monotherapy but associated with increased risk of gastrointestinal AEs and liver toxicity increased. Katchamart et al. [23] found the incidence of AEs was similar between the MTX plus LEF combination and MTX monotherapy groups. Similarly, in a retrospective study comparing MTX and LEF treatment with LEF monotherapy in RA patients, no significant difference was found in terms of effectiveness and AEs between both groups [24]. These findings suggest that while MTX plus LEF combination therapy may enhance treatment efficacy for MTX-refractory RA patients, it does not consistently increase the risk of AEs, underscoring its potential as a viable option for patients requiring intensified treatment.

In our study, monotherapy was preferred in elderly patients and a lower dose of LEF was preferred in the combined group. However, these regimes (combined treatment vs. LEF monotherapy) were not superior to each other in efficacy and AEs. In previous studies, AEs with LEF use were positively correlated with dosage [12]. Another study showed that LEF, when used at a low dose, is as effective as a high dose and has similar efficacy to MTX [25]. LEF monotherapy may be an alternative treatment to prevent AEs that may occur due to MTX misuse or renal impairment in elderly patients.

Badilla et al. [26] investigated the effectiveness of MTX and LEF combined in RA patients. They found that 83.5% of the patients continued this treatment, while AEs were reported in 25.6% of the patients. Two-thirds of these AEs are mild, and the problem is solved by reducing the dose of either medication. Half of the patients on combination therapy achieved remission or reduced RA activity with a significant steroid-sparing effect. In our study, a significantly reduction in steroid dose was observed in the combined use of MTX plus LEF compared to monotherapy in both RA and PsA patients.

In a study showing the effectiveness and safety of MTX and LEF combination therapy in RA patients, ACR 20 response was achieved by 71.6% of the study participants. In comparison, AEs occurred in 40.5% of the patients. Elevated liver enzymes were observed in 21.6% of the study participants, and a complete recovery was observed in most patients after discontinuing the drug [13]. In our study, Δ DAS28-CRP decreased significantly in patients who started the MTX plus LEF therapy compared to monotherapy. However, no difference was detected between MTX plus LEF treatment and monotherapy in terms of achieving disease remission. When multiple factors predicting LDA in RA patients were evaluated in multivariate regression analysis, combination therapy was not found to be independently associated with LDA, while baseline DAS-28 CRP was found to be an independent predictor. This may be due to the higher initial disease activity in patients receiving MTX and LEF combination therapy than in monotherapy.

In our study, AEs were observed in 19.2% of the RA combination group and liver enzyme elevation was detected in 11.5%. These values are similar to those in the monotherapy groups. The incidence of AEs could be caused by the dosage of the drugs in the combination group. These results highlight the need to closely monitor patients during treatment.

Lee et al. [13] found a higher ACR20 response in more than two-thirds of patients that were started on MTX and LEF simultaneously. Adding MTX and LEF treatment at different times and starting them simultaneously may affect the response to treatment [13].

Studies have also focused on the combined use of MTX plus LEF in PsA patients, but to a lesser extent than in RA. When MTX and LEF combined treatment was compared to MTX and placebo in PsA patients, MTX and LEF combined treatment was superior to MTX monotherapy in terms of effectiveness. In contrast, mild AEs were detected more in the combined group. The only significant difference in AEs between the two groups was a greater change in bowel habits in the combined group [27]. Approximately 15% of patients receiving MTX and LEF combination therapy used bDMARDs due to hepatotoxicity and ineffectiveness. Haroon et al. [28] reported that MTX and LEF combination therapy may be an effective treatment option for PsA. In our study, the rate of bDMARD or tsDMARD initiation in the group receiving combined treatment was 23.3%, and combined treatment was also found to be effective in the PsA group compared to monotherapy.

While the cumulative AEs of MTX and LEF monotherapies in PsA are similar to those of the combined use of these two drugs, combined treatment appears more effective.

Hypertension is a common AE associated with LEF. Hypertension is observed in 8.8% of patients, and it is important to monitor blood pressure in patients using LEF [29]. In our study, the cumulative incidence of new hypertension in RA patients with LEF monotherapy was 9.4% and was found to be significantly higher than in MTX monotherapy. In PsA patients, no difference in new hypertension development was observed between treatment groups. While some AEs associated with combination therapy may be severe enough to warrant discontinuation of the drugs, the majority are transient and manageable.

Hepatotoxicity is one of the most concerning potential AEs for the combination of MTX and LEF. Some studies have reported mild elevations in liver enzymes up to 45%, and it is known that <10% of patients have severe liver enzyme elevations that require discontinuation of the treatment and even death due to liver failure [30]. Although the reversibility of liver enzyme increases seen in current and previous clinical studies is reassuring, the potential for increased hepatotoxicity with the combination of MTX and LEF should be recognized, and the need for regular liver enzyme monitoring should be emphasized. It is important to individualize the treatment and adjust the dose, starting from a low dose.

Another important concern about MTX and LEF is hematological AEs, especially neutropenia and pancytopenia. Pancytopenia has been reported to occur in 1 in 4000 patients treated with LEF and in 1 in 575–822 patients with the combination of MTX and LEF [31]. However, one study states that the combined use of MTX and LEF is no different regarding hematological AEs compared to MTX monotherapy [32]. Similarly in our study, no difference was observed in terms of hematological AEs in the combined group of RA and PsA groups compared to monotherapy. This might be explained by the close and careful monitoring of patients for possible AEs in the MTX and LEF combination group.

Treatment discontinuation and AEs may be attributed to differences in individual patient and physician assessment of AEs, psychosocial factors, and patient education. Both patients’ and physicians’ perceptions of an AEs can be shaped by factors such as AE acceptance level, strategies to mitigate AEs, and the presence of alternative treatment options [33]. Educating patients on treatment benefits, risks, and procedures enhances compliance and improves health outcomes.

One strength of our study is that the safety and efficacy of MTX and LEF combination therapy was evaluated with a relatively large cohort of RA and PsA patients. However, our study was limited because it was a retrospective, single-center study, and therefore, no follow-up was made in terms of AEs. Due to the retrospective nature of the study, randomization was not specifically applied to treatment groups. This design limits the ability to establish causal relationships and may introduce selection bias, which could confound the observed outcomes. Another limitation was that no data was collected on patient adherence to medication or whether the medication was temporarily or permentantly discontinued due to AEs. The lack of long-term safety data on AEs may lead to an incomplete understanding of the risk profile of the treatment, highlighting the need for extended follow-up periods in future research. Finally, none of the patients started with LEF plus MTX combined. A prospective, randomized controlled trial would provide more robust evidence regarding the efficacy and safety of the intervention, addressing these limitations.

In conclusion, when the treatment is individualized with the appropriate dose for a patient, the regimens are not superior to each other in terms of AEs. MTX plus LEF may be more effective in reducing initiation b/tsDMARD and steroid doses when used together than alone. This research provides promising insights into utilizing MTX plus LEF for patients with RA and PsA. Future prospective multicenter randomized controlled studies following up AEs in the long-term should be performed. Also, to determine true differences between regimes, noninferiority or equivalence study designs should be preferred.

## Figures and Tables

**Figure:**
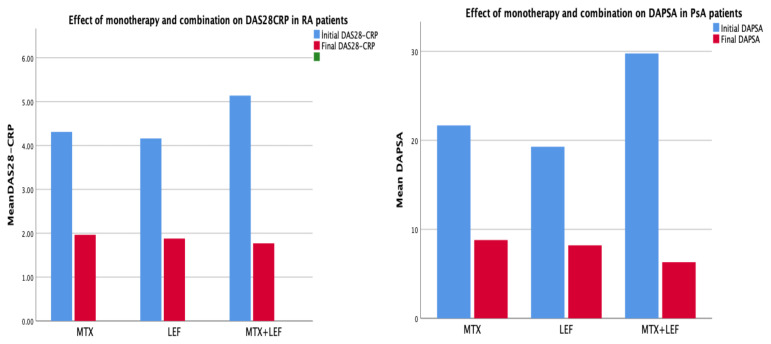
Effect of monotherapy and combination on DAS28CRP and DAPSA in RA and PsA patients.

**Table 1:** Comparison of demographic, clinical, and activity characteristics of RA and PsA patients.

Variables	RA (n = 385)	PsA (n = 143)	p
**Age, years, mean (SD)**	55.7 (12.3)	48.7 (14.2)	**<0.001**
**Age at diagnosis, years, mean (SD)**	49.5 (12.4)	45.1 (14.5)	**<0.001**
**Sex female, n (%)**	301 (78.2)	108 (75.5)	0.516
**Disease duration, years, median (IQR**)	3.5 (6)	3 (4)	0.907
**Follow-up period, months, mean (SD)**	29.4 (10.4)	27.3 (11.5)	0.752
**Smoking status current, n (%)**	125 (32.5)	61 (42.7)	**0.029**
**At least one comorbidity, n (%)**	241 (62.6)	67 (46.9)	**0.001**
**MTX dosage, mg/week, mean (SD)**	15.5 (2.72)	15.4 (2.74)	0.752
**LEF dosage, mg/week, mean (S**D)	131.9 (21.7)	130 (24.6)	0.564
**Prednisolone dosage, mg/day, mean (S**D)	5.6 (1.4)	5.8 (1.4)	0.421
**MTX and LEF combination, n (%)**	52 (11.5)	30 (21)	**0.035**
**MTX adverse effects, n (**%)	70/347 (20.2)	22/128 (17.2)	0.465
**LEF adverse effects, n (%)**	25/134 (18.7)	11/59 (18.6)	0.998
**Adverse effects n (%)**	93 (24.2)	31(21.7)	0.551
**Serious adverse effects, n (%)**	3 (0.8)	2 (1.4)	0.805

Bold values indicate statistically significant differences (p < 0.05).

**Abbreviations:** RA: Rheumatoid arthritis, PsA: Psoriatic arthritis, MTX: Methotrexate, LEF: Leflunomide, DAPSA: Disease Activity in Psoriatic Arthritis.

**Table 2:** Comparison of MTX, LEF monotherapy, and combination therapy in RA in terms of effectiveness.

Variables	MTX monotherapy (n = 248)	LEF monotherapy (n = 85)	MTX and LEF (n = 52)	p
**Age, years, mean (SD)**	53.8 (12.4)	63.4 (14.2)	55.7 (12.3)	**<0.001**
**Age at diagnosis, years, mean (SD)**	48.9 (13.1)	57.4 (14)	49.5 (12.4)	**<0.001**
**Sex female, n (%)**	205 (82.7)	61 (71.8)	35 (67.3)	**0.014**
**Disease duration, years, median (IQR)**	2 (5)	3 (8)	3.5 (6)	0.140
**Follow-up period, months, mean (SD)**	25.8 (11.3)	25.9 (10.7)	29.5 (10.4)	0.051
**Smoking status current, n (%)**	72 (29)	21 (24.7)	32 (61.5)	**<0.001**
**At least one comorbidity, n (%)**	155 (62.5)	54 (63.5)	32 (62.5)	0.972
**Seropositivity, n (%)**	162 (65.3)	51 (60)	36 (69.2)	0.514
**Initial prednisolone dosage, mg, mean (SD)**	5.49 (1.25)	5.82 (1.59)	6.32 (2.17)	**0.001**
**Final prednisolone dosage, mg, mean (SD)**	4.36 (1.57)	4.8 (1.26)	3.36 (2.66)	**<0.0**01
**Δ prednisolone dosage, mg, mean (SD**)	1.12 (1.5)	0.93 (1.47)	2.95 (2.86)	**<0.001**
**MTX dosage, mg/week, mean (SD)**	14.77 (1.38)	-	14.6 (2.94)	**0.044**
**LEF dosage, mg/week, mean (SD)**	-	137.3 (13.7)	114.8 (32.3)	**<0.001**
**Initial DAS28-CRP, mean (SD)**	4.71 (0.62)	4.59 (0.66)	5.15 (1.14)	**<0.001**
**Initial DAS active disease (≥ 3.2), n (%)**	238 (96)	78 (91.8)	52 (100)	0.066
**Final DAS28-CRP, mean (SD)**	2.19 (0.62)	1.88 (0.45)	1.78 (0.57)	0.169
**Final DAS active disease (≥ 3.2), n (%)**	12 (4.8)	3 (3.5)	1 (1.9)	0.806
**Δ DAS28-CRP, mean (SD)**	2.51 (0.74)	2.71 (0.71)	3.36 (0.88)	**<0.001**
**bDMARD initiation, n (%)**	85 (34.3)	29 (34.1)	9 (17.3)	0.061
**tsDMARD initiation, n (%**)	65 (24.2)	14 (16.4)	6 (11.5)	**0.028**
**b/ts DMARD initiation, n (%)**	149 (60.1)	43 (50.6)	15 (28.9)	**<0.001**

Bold values indicate statistically significant differences (p < 0.05).

**Abbreviations:** RA: Rheumatoid arthritis, MTX: Methotrexate, LEF: leflunomide, DAS28-CRP: The Disease Activity Score C-Reactive Protein, Δ DAS28-CRP: Decrease in DAS-28 CRP score from baseline to last visit, Δ prednisolone: Decrease in prednisolone score from baseline to last visit, bDMARD: Biological disease-modifying antirheumatic drugs, tsDMARD: Targeted synthetic disease-modifying antirheumatic drug.

**Table 3:** Comparison of MTX, LEF monotherapy, and combination therapy in PsA in terms of effectiveness.

Variables	MTX monotherapy (n = 83)	LEF monotherapy (n = 30)	MTX and LEF (n = 30)	p
**Age, years, mean (SD)**	42.3 (8.6)	53.3 (12.9)	48.7 (14.2)	**0.001**
**Age at diagnosis, years, mean (SD)**	36.2 (10.3)	45.4 (15.1)	45.1 (14.5)	**0.006**
**Sex, female, n (%)**	64 (77.1)	22 (73.3)	22 (73.3)	0.895
**Disease duration, years, median (IQR)**	3 (5)	4 (13)	3 (4)	0.582
**Follow-up period, months, mean (SD)**	24 (12)	24 (12)	36 (21)	0.373
**Smoking status current, n (%)**	38 (45.8)	6 (20)	17 (56.7)	**0.011**
**At least one comorbidity, n (%)**	33 (39.8)	20 (66.7)	14 (46.7)	**0.041**
**Initial prednisolone dosage, mg, mean (SD)**	5.44 (1.03)	5.62 (1.1)	6.91 (1.93)	**<0.001**
**Final prednisolone dosage, mg, mean (SD)**	3.92 (1.61)	3.79 (1.75)	0.45 (0.76)	**<0.001**
**Δ prednisolone, mg, mean (SD)**	1.51 (1.6)	1.83 (2.19)	6.45 (1.57)	**<0.001**
**MTX dosage, mg/week, mean (SD)**	15 (2.04)	-	14.1 (3.2)	**0.002**
**LEF dosage, mg/week, mean (SD)**	-	140 (0)	116.6 (33.5)	**<0.0**01
**Initial DAPSA, mean (SD)**	25.18 (8.9)	21.36 (5.5)	29.76 (5.18)	**<0.001**
**Initial DAPSA active disease (≥ 15), n (%)**	78 (94)	25 (86.2)	29 (100)	0.087
**Final DAPSA, mean (SD)**	10.9 (5.17)	10 (5.9)	6.31 (2.68)	**0.027**
**Final DAPSA active disease (≥ 15), n (%)**	7 (8.4)	3 (10.3)	1 (3.4)	0.628
**Δ DAPSA, mean (SD)**	14.2 (7.13)	11.36 (3.44)	23.45 (5.18)	**<0.001**
**bDMARD initiation, n (%)**	53 (63.8)	14 (46.6)	7 (23.3)	**0.001**
**tsDMARD initiation, n (%**)	4 (4.7)	0 (0)	0 (0)	0.246
**b/tsDMARD initiation, n (%)**	57 (68.6)	14 (46.6)	7 (23.3)	**<0.001**

Bold values indicate statistically significant differences (p < 0.05).

**Abbreviations:** PsA: Psoriatic arthritis, MTX: Methotrexate, LEF: Leflunomide, DAPSA: Disease Activity in Psoriatic Arthritis, Δ DAPSA: Decrease in DAPSA score from baseline to last visit, Δ prednisolone: Decrease in prednisolone score from baseline to last visit, bDMARD: Biological disease-modifying antirheumatic drugs, tsDMARD: Targeted synthetic disease-modifying antirheumatic drugs.

**Table 4:** Comparison of MTX, LEF monotherapy, and combination therapy in RA in terms of AEs.

Variable	MTX monotherapy n = 248	LEF monotherapy n = 85	MTX and LEF n = 52	p-value MTX vs LEF	p-value MTX vs MTX+LEF	p-value LEF vs MTX+LEF	Confidence interval (CI)		
							MTX monotherapy n = 248	LEF monotherapy n = 85	MTX and LEF n = 52
**MTX-related adverse effect**	59 (23.8)	-	11 (13.5)		0.857		(0.19, 0.29)		(0.12, 0.34)
**LEF-related adverse effect**	-	20 (23.5)	5 (10.2)			0.043		(0.16, 0.34)	(0.04, 0.21)
**Adverse effects**	59 (23.8)	24 (28.2)	10 (19.2)	0.468	0.588	0.309	(0.19, 0.29)	(0.2, 0.39)	(0.11, 0.32)
**Serious adverse effects**	1 (0.4)	1 (1.2)	1 (1.9)	0.446	0.317	1.0	(0.0, 0.02)	(0.0, 0.06)	(0.0, 0.1)
**Gastrointestinal adverse effects**	32 (9.2)	9(10.6)	2 (3.8)	0.703	0.089	0.206	(0.09, 0.18)	(0.06, 0.19)	(0.01, 0.13)
**Hepatotoxicity**	17 (6.9)	6 (7.1)	6 (11.5)	1.0	0.254	0.371	(0.04, 0.11)	(0.03, 0.15)	(0.05, 0.23)
**Pulmonary adverse effect**	4 (1.6)	1 (1.2)	0	1.0	1.0	1.0	(0.01, 0.04)	(0.0, 0.06)	(0.0, 0.07)
**Gynecological adverse effect**	3 (1.2)	0	1 (1.9)	0.573	0.535	0.38	(0.0, 0.03)	(0.0, 0.04)	(0.0, 0.1)
**Neurological adverse effect**	1 (0.4)	4 (4.7)	2 (3.8)	0.016	0.079	1.0	(0.0, 0.02)	(0.02, 0.11)	(0.01, 0.13)
**Hematological adverse effect**	7 (2.8)	5 (5.9)	2 (3.8)	0.192	0.657	0.709	(0.01, 0.06)	(0.03, 0.13)	(0.01, 0.13)
**Dermatological adverse effect**	9 (3.6)	4 (4.7)	2 (3.8)	0.746	1.0	1.0	(0.02, 0.07)	(0.02, 0.11)	(0.01, 0.13)
**Newly developed hypertension**	4 (1.6)	8 (9.4)	3 (5.7)	**0.003**	0.103	0.533	(0.01, 0.04)	(0.05, 0.17)	(0.02, 0.16)

Bold values indicate statistically significant differences (p < 0.05).

**Abbreviations:** MTX: Methotrexate, LEF: Leflunomide.

**Table 5:** Comparison of MTX, LEF monotherapy, and combination therapy in PsA in terms of AEs.

Variables	MTX monotherapy (n = 83)	LEF monotherapy (n = 30)	MTX and LEF (n = 30)	p-value MTX vs LEF	p-value MTX vs MTX+LEF	p-value LEF vs MTX+LEF	Confidence interval (CI)		
							MTX monotherapy n=83	LEF monotherapy n=30	MTX and LEF n=30
**MTX-related adverse effect, n (%**)	15 (18.1)		7 (23.3)		0.593		(0.1, 0.28)	(0, 0.12)	(0.1, 0.42)
**LEF-related adverse effect, n (%)**	-	6 (20)	5 (17.2)			1.0	(0, 0.04)	(0.08, 0.39)	(0.06, 0.35)
**Adverse effects, n (%)**	15 (18.1)	9 (30)	7 (23.3)	0.197	0.593	0.771	(0.1, 0.28)	(0.15, 0.49)	(0.1, 0.42)
**Serious adverse effects, n (%)**	0 (0)	1 (3.3)	1 (3.3)	0.265	0.265	1.0	(0, 0.04)	(0.0, 0.17)	(0.0, 0.17)
**Gastrointestinal adverse effects, n (%)**	6 (7.2)	2 (6.7)	3 (10)	1.0	0.697	1.0	(0.03, 0.15)	(0.01, 0.22)	(0.02, 0.27)
**Hepatotoxicity n (%)**	4 (4.8)	1 (3.3)	5 (16.7)	1.0	0.054	0.195	(0.01, 0.12)	(0.0, 0.17)	(0.06, 0.35)
**Pulmonary adverse effect, n (%)**	0 (0)	2 (6.7)	0 (0)	0.069	1.0	0.492	(0, 0.04)	(0.01, 0.22)	(0, 0.12)
**Gynecological adverse effect, n (%)**	1 (1.2)	0 (0)	0 (0)	1.0	1.0	1.0	(0.0, 0.07)	(0, 0.12)	(0, 0.12)
**Neurological adverse effect, n (%)**	1 (1.2)	1 (3.3)	0 (0)	0.462	1.0	1.0	(0.0, 0.07)	(0.0, 0.17)	(0, 0.12)
**Hematological adverse effect, n (%)**	4 (4.8)	3 (10)	1 (3.3)	0.38	1.0	0.612	(0.01, 0.12)	(0.02, 0.27)	(0.0, 0.17)
**Dermatological adverse effect, n (%)**	3 (3.6)	2 (6.7)	0	0.607	0.564	0.492	(0.01, 0.1)	(0.01, 0.22)	(0, 0.12)
**Newly developed hypertension, n (%)**	2 (2.4)	2 (6.6)	1 (3.3)	0.286	1.0	1.0	(0.0, 0.08)	(0.01, 0.22)	(0.0, 0.17)

**Abbreviations:** MTX: Methotrexate, LEF: Leflunomide.

**Table 6:** Evaluation of factors contributing to DAS28-CRP LDA in RA in univariate and multivariate regression analysis.

Predictors	Univariate		Multivariate	
	OR (95% CI)	p	OR (95% CI)	p
**Combination vs monotherapy**	1.07(0.16–4.08)	0.931	0.48(0.02–3.11)	0.518
**Age, years**	1.00(0.97–1.05)	0.808		
**Sex, female vs male**	0.96(0.21–3.16)	0.952		
**Disease duration, years**	1.04(0.98–1.10)	0.160	1.01(0.91–1.11)	0.775
**Follow-up period, months**	0.98(0.94–1.03)	0.455		
**Smoking vs not smoking**	1.68(0.54–4.95)	0.345		
**At least one comorbidity vs any comorbidity**	3.47(0.93–22.53)	0.107		
**Initial prednisolone dosage, mg/day**	1.22(0.89–1.58)	0.167		
**MTX dosage, mg/week**	1.25(1.03–1.50)	**0.020**	1.27(1.02–1.59)	**0.031**
**Initial DAS28-CRP**	3.66(2.09–7.15)	**<0.001**	4.38(2.14–10.90)	**<0.001**
**b/tsDMARD (initiation vs not initiation)**	5.42(1.45–35.11)	**0.028**		

Bold values indicate statistically significant differences (p < 0.05).

**Abbreviations:** OR: Odds ratio, CI: Confidence interval, DAS28-CRP: The Disease Activity Score C-Reactive Protein, LDA: Low disease activity, b/tsDMARD: Biological/targeted synthetic disease-modifying antirheumatic drugs.

**Table 7:** Evaluation of factors contributing to DAPSA LDA in PsA in univariate and multivariate regression analysis.

Predictors	Univariate		Multivariate	
	OR (95% CI)	p	OR (95% CI)	p
**Combination vs monotherapy**	0.38 (0.02–2.15)	0.370	0.37 (0.02–4.39)	0.443
**Age, years**	1.01 (0.96–1.06)	0.638		
**Sex, female vs male**	1.41 (0.29–5.41)	0.633		
**Disease duration, year**	1.14 (1.03–1.26)	**0.008**	1.17 (1.02–1.38)	**0.039**
**Follow-up period, months**	0.99 (0.94–1.05)	0.735		
**Smoking vs not smoking**	2.13 (0.58–8.65)	0.259		
**At least one comorbidity vs any comorbidity**	1.77 (0.48–7.20)	0.393		
**Initial prednisolone dosage, mg**	1.24 (0.80–1.79)	0.286		
**Initial DAPSA**	1.50 (1.25–1.96)	**<0.001**		
**b/tsDMARD (initiation vs not initiation)**	0.82 (0.22–3.08)	0.765		

Bold values indicate statistically significant differences (p < 0.05).

Abbreviations: PsA: Psoriatic arthritis, DAPSA: Disease Activity in Psoriatic Arthritis, LDA: Low disease activity, bDMARD: Biological disease-modifying antirheumatic drugs.
